# Dual mobility total hip arthroplasty in hemiplegic patients

**DOI:** 10.1051/sicotj/2017024

**Published:** 2017-06-02

**Authors:** Ayman T. Henawy, Ahmed Abdel Badie

**Affiliations:** 1 Department of Orthopedic Surgery and Trauma, Suez Canal University Hospitals Kilo 4.5 Ring Road 41111 Ismailia Egypt

**Keywords:** Total hip replacement, Hemiplegia, Dual mobility cups, Stability, Unconstrained tripolar

## Abstract

*Introduction:* The rate of cerebrovascular insults is increasing, currently leaving many patients with difficulties to maintain their balance due to muscular weakness and/or poor central control. Those patients are at risk of dislocation when total hip arthroplasty (THA) is planned. Instability remains the most significant issue after primary THA especially in such groups of patients. The risk is more pronounced when other factors are added such as, older age, femoral neck fractures, avascular necrosis and/or hip osteoarthritis. Dual mobility cup (DMC) is considered as a prosthesis with higher inherent stability that may help in such situation. In this patient series, we aimed to evaluate stability, clinical and radiological results of dual mobility THA done on the weak limb of hemiplegic patients.

*Methods*: Twenty-four consecutive hemiplegic patients have undergone DMC with a mean age of 68 years. The indication for surgery was hip osteoarthritis in one third of the patients and femoral neck fractures in the remaining patients. Those patients were capable of walking prior to hospital admission despite weakness. Those patients were observed postoperatively for at least one year. Clinical results and complications were recorded.

*Results*: After a minimum of one year, 91.6% of the patients have satisfactory results. No cases of hip or intraprosthetic dislocation were observed.

*Discussion*: Dual mobility THA in the hemiplegic patients provides both efficacy and stability with good functional results.

## Introduction

With improvement of health care standards, more attention should be directed to the handicapped group of population, especially with increased rate of intracranial hemorrhage and stroke hemiplegia (sometimes called hemiparesis). It is caused by irreversible injury to parts of the brain that control movements of the contralateral limbs, trunk, face, etc. People with hemiparesis often have difficulties to keep their balance due to limb weaknesses leading to an inability to shift their body properly. Hemiparesis with origin in the lower section causes loss of both gross and fine motor skills. Pure motor hemiparesis, a form of hemiparesis characterized by sided weakness in the leg, arm, and face, and is the most commonly diagnosed form of weakness in the leg, arm, and face, is the most commonly diagnosed form of hemiparesis [1].

Several kinds of problems may happen after THA in hemiplegic patients. The most common one is the instability. The causes for instability can be classified into surgery-related, patient- and implant-related, for example dislocation is more common in females or people with a prior hip surgery or neuromuscular conditions that lead to weak hip muscles [2].

Dual mobility cups (DMCs) (also known as unconstrained tripolar implants) are indicated in THA when the risk of instability is great especially in patients with weak muscles. The incidence of instability after standard THA in the primary setting has been reported to be as high as 7% [3, 4], with revision rates up to 22.5% of all THA revisions in the United States [5]. Readmission and prosthetic revision surgery carry considerable economic cost, a cost raise of 148% is anticipated when prosthetic revision is considered [6]. Modifications in surgical technique (e.g., anterior surgical approach, repair of posterior soft tissues, increasing offset, and improvement of abductor tension) and the use of larger size femoral heads with greater inherent stability decrease the risk of instability after THA. DMCs have recently gained wider attention as a better option in preventing and treating instability in THA along with keeping satisfactory clinical outcomes and implant survival [7–11].

The concept of dual mobility is based on the fact that simple mobility implants with small head can dislocate too early when the femoral neck comes bumping into the acetabular cup’s rim. The dual mobility implant keeps the small head stemming from the Charnley’s hip prosthesis to have little wear but an added polyethylene (PE) insert acts as a big femoral ball and allows a wider range of motion. The femoral head slides against the inner surface and the outer surface slides against the metallic shell. This concept has two main advantages: a great range of motion and an outstanding stability because it delays lever-out dislocation. It was proposed that the use of DMCs can improve the abduction/adduction up to 126°, 186° for the flexion/extension and 220° for rotation [12]. Studies showed that the dislocation risk index decreased by using the DMCs when compared to other constrained tripolar or a bipolar systems. This concept is very promising in THA in weak musculature [13–15].

## Patients and methods

A prospective study was carried out between February 2013 and June 2015 on 24 hemiplegic patients (14 males and 10 females) treated by primary THAs done for the weak limb using DMCs. The mean age was 68 years (range, 53–79 years). The mean body mass index (BMI) was 26 (range, 20–32). The indication for surgery was femoral neck fractures in 16 hips (67%). The remaining eight hips (33%) were divided into primary osteoarthritis in two cases, previous pediatric hip disorder in two other cases, and four cases of femoral head osteonecrosis. These conditions may jeopardize the risk of instability due to neuromuscular disease (Table 1). The mean time passed after stroke or cerebral hemorrhage was 30 months (range, 6–67). Those patients were capable of independent walking prior to surgery. In some patients, we needed to hospitalize them for a week preoperatively to correct the coagulation problems as they were kept on oral anticoagulants and anti-platelets.

**Table 1.** Indications for surgery in the study group.

Etiology	No	%
Femoral neck fractures	16	67
Primary arthritis	2	8.4
Arthrosis secondary to childhood disease	2	8.4
Arthrosis secondary to avascular necrosis	4	17

**Figure 1.**
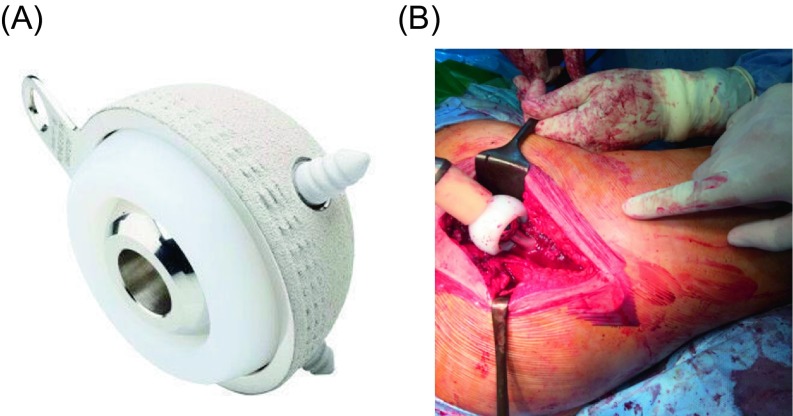
(A) Novae^®^ cup used in the cases, a tripod cup that contain a superior fixation screw and two anchorage studs, (B) intraoperative photo during impaction of the PE liner over 22.5 mm head prior to reduction.

**Figure 2.**
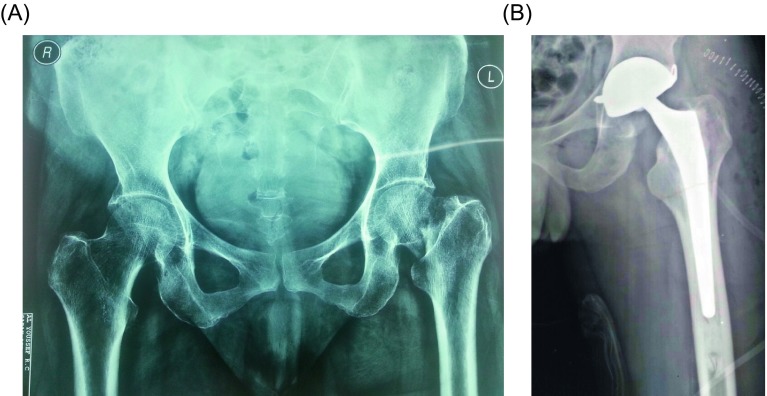
(A) Preoperative radiography of a 65 years old female patient with a fracture of the left femoral neck, (B) postoperative X-ray of a cemented dual mobility THA, the superior fixation screw was left due to the press fit metal cup.

**Figure 3.**
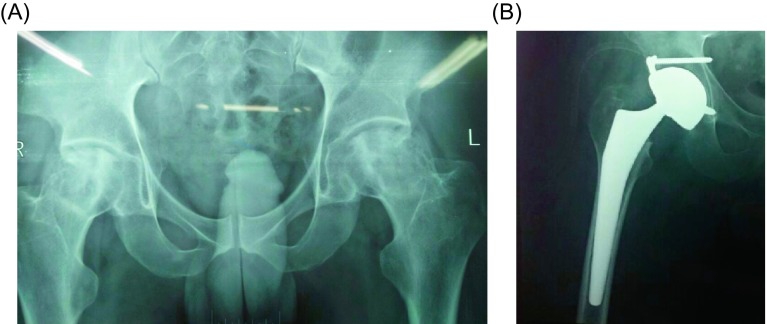
(A) Preoperative X-ray of a 57 years old male patient with bilateral hip arthritis due to AVN, (B) postoperative X-ray of the patient after cementless dual mobility THA with a good position.

We used Novae^®^ cups (Figure 1A, 1B), a product of Serf Company. They provide a wide range of sizes of cups starting from 45 to 61 mm. The cup is sprayed with titanium to enhance primary fixation and hydroxyapatite coated to allow osteointegration. Polyethylene (PE) liners are available in two sizes 22.2 and 28 mm. The PE used is dense ultra-high molecular weight UHMWPE that provides less wear with viscoelastic properties. The femoral neck component is smooth with no sharp edges, skirts nor impaction holes. Two types of components were implanted: 18 (75%) were cemented (Figures 2A, 2B), and six (25%) were cementless (Figures 3A, 3B). Stems were made of PRO^®^ titanium, a product of Serf Company, with a neck diameter of 13 mm.

### Surgical procedure

We used the lateral transgluteal approach for all patients under epidural anesthesia. After reaming down to bleeding bone, a cup trial component of appropriate size was inserted anatomically, without impaction, blocked at the edge to provide a room for impaction behind the dome. The mean cup diameter was 55 mm (range, 46–61 mm). The head diameter (cobalt chrome) was 22.5 mm in 20 cases and 28 mm in four cases.

### Postoperative management and follow-up

Full weight bearing was permitted the day after surgery when cemented stems were used and delayed to six weeks when the cementless stems were utilized. We used suction drains for all patients; all drains were removed before day three postoperatively. The patients were kept on low molecular weight heparin for 40 days.

The 24 patients were reviewed prospectively with a minimum follow-up period of 12 months (range 12–18). All patients were recalled specifically for this study, and they underwent clinical and radiographic assessment.

Clinical evaluation using the Merle d’Aubigné hip score [16], and the Harris hip score [17], was used for assessment in the follow-up visits. In addition, because of large-diameter articulations, we looked for iliopsoas irritation at follow-up examination (for each patient complaining of groin pain).

Radiographs (anteroposterior (AP) pelvis) were taken at follow-up. We compared immediate postoperative radiographs with follow-up radiographs and radiographs at last follow-up to evaluate cup fixation according to Massin et al. [18]. Loosening was diagnosed if the cup migration exceeded 5 mm or 5° in any direction and osteolysis is defined as a new or expanding darkened area in which no trabeculae were visible adjacent to the prosthetic components. If osteolysis presents, the lesions were classified according to Delee and Charnley [19] for the acetabulum and Gruen et al. for the femur [20].

The cup inclination angle was measured with respect to the horizontal line joining the teardrops on AP views. The cup center position was determined on AP pelvis views using a template for each side: the *x*-axis represents the horizontal tear-drop line and the *y*-axis represents the vertical line through the teardrop. The healthy side was considered as an anatomic reference position used for comparison and detection of deviation. The cup stability was assessed on an AP pelvis view with constant 110% magnification, measuring the vertical distance between the cup center and the tear-drop line, following. The bone/implant interface was examined for radiolucency at the cup edge, not seen on the immediate postoperative image, in the three Charnley zones [19].

## Results

The operative time ranged from one and half hours to three hours with a mean blood loss of 673 cc. All the patients were discharged on day three postoperatively except for four patients who needed three more days due to suspected deep vein thrombosis (DVT) in two patients and for control of blood sugar in the other two (Table 2). Clinical data were collected through the follow-up period. We had two patients with late postoperative complications, one case developed DVT five months post-operatively for which he was readmitted and treated. One case had late infected arthroplasty after eight months, which was managed by extraction of the implant and antibiotic spacer. We had two mortalities (one patient died five months and the other six months postoperatively due to unrelated medical causes).

**Table 2.** Operative data and length of hospital stay.

Variables	No	%
Hospital stay		
Three days	20	83
More than three days	4	17
Operative time		
Less than 2 h	16	67
2–3 h	8	33
Intra op bleeding		
Less than 500 cc	10	42
500–1000 cc	14	58

The mean Merle d’Aubigné functional score rose (*p* < 0.001) from 8 (range, 0–16) to 17 (range, 14–18). The mean Harris hip score increased (*p* < 0.001) from 36 (range, 0–73) preoperatively to 94 (range, 88–100) at last follow-up. At the last follow-up visit, the 21 patients had no pain or infrequent and mild pain.

Radiographs (AP pelvis) were taken at follow-up and assessed by the single observer conducting the clinical examination. We compared immediate postoperative radiographs with intermediate radiographs and radiographs at the last follow-up. There were no dislocations either of the large-articulation or intraprosthetic dislocation.

The absence of radiolucent spaces, denoting osteolysis, was seen in 91.6% of cases (20 out of the 24 patients at final follow-up). The cup incorporation with native bone was seen in two more patients who had radiolucent zones at the initial follow-up. Only two patients had radiolucent space less than 1 mm at the final follow-up. The cup inclination angle was between 40° and 45° in 20 patients (83%). Postoperative X-ray comparison in the 24 cases with the healthy contralateral side showed a mean 3 mm (range 1–6 mm) medialization and 3 mm (0–6 mm) ascension of the hip. The dome of the cup crossed the iliosciatic line (protrusion) in one case. The patient with poor cup fixation was 65 years old with follow-up and preparation for revision but the general condition did not allow for revision surgery and he died after six months postoperatively.

No patients showed signs of loosening at the final follow-up visit.

## Discussion

Instability remains a significant issue after both primary and revision THA. This complication is more likely in patients with neurological deficit such as hemiplegia where patients have weak muscles. Dual mobility components, which were initially designed in 1975 to provide a satisfactory wider hip range of motion, can also provide a viable alternative in preventing and treating instability. Most series of DM cups encountered no large-articulation dislocation [2–21], while others report a rate of dislocation of only about 1% [22]. This shows that the DM cups are very stable with very rare large-articulation dislocation events [22–24].

The main result of this study was a no large-articulation dislocation rate (0%), that was below the rates reported with conventional THA with fixed polyethylene done for patients without neurological compromise (ranging from 1.8% to 2.9% at five years follow-up). Compared to other series of DM cups, this study indicated the actual large-articulation dislocation previously considered to be zero.

Intraprosthetic dislocation is a specific complication of DMCs. The head extracts from the polyethylene indicating retention failure between the head and polyethylene in small articulations [25]. Intraprosthetic dislocations were more frequent, ranging from 2% to 4% [8–26]. This complication is minimized in the newer designs that utilize smooth necks avoiding a rough neck surface, the presence of skirts on the femoral side and the use of a dense polyethylene (PE) insert on the acetabular side [25]. We did not encounter a single case of intraprosthetic dislocation maybe due to the use of denser UHMWPE decreasing the risk of osteolysis and a friendly smooth neck preventing friction. However, we can say that this problem can be anticipated with a longer term follow-up.

The only difference in the rehabilitation of those patients was the ipsilateral weakness of the upper limbs, which is a problem in initiating partial weight bearing. The use of frame walkers and crutches was difficult in those patients. We considered this fact in deciding whether to cement those patients or not. We have chosen the cementless components only for the patients with better upper extremity control aiding the weight bearing.

Our functional results are comparable with those of conventionally fixed cementless polyethylene components done for healthy individuals without any neurological deficit. DM cups behave like large-head THAs, groin pain is not an uncommon adverse event. Despite this fact, our incidence of groin pain was 0%, far below the rates with large-diameter heads and hip resurfacing [27, 28].

Other options that may be used when the risk of dislocation is high include constrained cups and tripolar constrained THA. Lefevre et al., described a cemented retentive cup for cases who have grossly deficient soft tissue and patients with neurologic deficiencies. They include 73 patients with primary THA. They observed 97% stable hips but, progressive radiolucent lines developed with a rate of 17.6% with an average time of onset of 36.3 months postoperatively [29]. Almost similar rates of radiolucent lines and loosening were observed by many authors [30–32].

However, those results were based mainly on revision cases. Hernigou et al., published a retrospective study of primary THA in neurologically affected patients. They conclude that the use of a retentive liner improved the midterm survival of THA from 82% to 98% [33].

These higher rates of stability were arguable. Although constrained liners are intended to stabilize the hip by mechanically preventing dislocation, the resulting loss of range of motion may lead to impingement and, ultimately, to implant failure in a rate that ranges from 40% to 100%. The proposed mechanisms of failure are various with nearly a constant finding of impingement damage of the rim of the PE liner. The durability of these devices is unlikely to improve unless the mechanical demands are modified through an increased range of motion leading to less frequent rim impingement [34].

The DMC is not a mechanical blocker of dislocation. There is no stress on the PE rim that limits the range of motion to prevent dislocation before its failure. The DMC appears more stable with providing a wider range of motion with avoiding impingement of the rim with less wear and failure rates.

Based on our findings, we recommend DM cups for all patients with a higher risk of dislocation. The DM cups may give an answer to the instability issues in the patients with weaker neuromuscular control (Parkinson’s disease, dementia, palsy), who are at high risk of dislocation. Also, in patients with residual poliomyelitis, DM cups can be considered but they need smaller sizes as their hips do not grow to larger sizes as a result of the early onset of the pathologic insult.

There are strengths and limitations to our study. Our study was done on a group of patients that present frequently in all communities. The incidence of cerebrovascular stroke is increasing. Standard follow-up protocol was used. Outcome instruments were objective and widely used, and we analyzed both patient and physician-assessed outcome measures.

As for limitations, the sample size of the study was small; hence, statistical power to detect subgroup differences was limited. Secondly, we lacked information about socio-economic status and functional demands of patients. Thirdly, exclusion of septic hips would have yielded slightly better results. Fourthly, the short-term follow-up may decrease the incidence of complications that may develop later as intraprosthetic dislocation.

Dual mobility cups were efficient in reducing THA dislocations in hemiplegic patients who are at more risk of instability due to neurologic impairment. Furthermore, they gave good functional results.

## Conflict of interest

AH and AA certify that they have no financial conflict of interest (e.g., consultancies, stock ownership, equity interest, patent/licensing arrangements, etc) in connection with this article.

## References

[1] Rowland LP (2000) Syndromes caused by weak muscles, in: Merritt’s neurology, 10th edn, Rowland LP, Editor Philadelphia, USA, Lippincott Williams & Wilkins Publishers.

[2] Guyen O, Pibarot V, Vaz G, Chevillotte C, Carret JP, Bejui-Hugues J (2007) Unconstrained tripolar implants for primary total hip arthroplasty in patients at risk for dislocation. Arthroplasty J 22(6), 850–858. 10.1016/j.arth.2006.11.014 17826276

[3] Huten D, Langlais F (2012) Luxations et subluxations des prothèses totales de hanche [Dislocation and subluxation after total hip replacement], in 13 mises au point en chirurgie de la hanche. Huten D, Editor Paris, Elsevier Masson, 118–164.

[4] Kershaw CJ, Atkins RM, Dodd CA, Bulstrode CJ (1991) Revision total hip arthroplasty for aseptic failure. A review of 276 cases. J Bone Joint Surg Br 73, 564–568. 2071636 10.1302/0301-620X.73B4.2071636

[5] Massin P, Tanaka C, Huten D, Duparc J (1998) Treatment of aseptic acetabular loosening by reconstruction combining bone graft and Muller ring. Actuarial analysis over 11 years. Rev Chir Orthop Reparatrice Appar Mot, 84, 51–60. 9775022

[6] Berry DJ, von Knoch M, Schleck CD, Harmsen WS (2004) The cumulative long-term risk of dislocation after primary Charnley total hip arthroplasty. J Bone Joint Surg Am 86-A, 9–14. 14711939 10.2106/00004623-200401000-00003

[7] Caton JH, Prudhon JL, Ferreira A, Aslanian T, Verdier R (2014) A comparative and retrospective study of three hundred and twenty primary Charnley type hip replacements with a minimum follow up of ten years to assess whether a dual mobility cup has a decreased dislocation risk. Int Orthop 38(6), 1125–1129. 24737147 10.1007/s00264-014-2313-2 PMC4037498

[8] Philippot R, Camilleri JP, Boyer B, Adam P, Farizon F (2009) Theuse of a dual-articulation acetabular cup system to prevent dislocationafter primary total hip arthroplasty: analysis of 384 cases at a meanfollow-up of 15 years. Int Orthop 33, 927–932. 18521598 10.1007/s00264-008-0589-9 PMC2899002

[9] Prudhon JL, Ferreira A, Verdier R (2013) Dual mobility cup: dislocation rate and survivorship at ten years of follow-up. Int Orthop 37, 2345–2350. 24026216 10.1007/s00264-013-2067-2 PMC3843189

[10] Combes A, Migaud H, Girard J, Duhamel A, Fessy MH (2013) Low rate of dislocation of dual-mobility cups in primary total hip arthroplasty. Clin Orthop Relat Res 471(12), 3891–3900. 23516032 10.1007/s11999-013-2929-3 PMC3825881

[11] Epinette JA, Béracassat R, Tracol P, Pagazani G, Vandenbussche E (2013) Are modern dual mobility cups a valuable option in reducing instability after primary hip arthroplasty, even in younger patients? J Arthroplasty 29(6), 1323–1328. 24444567 10.1016/j.arth.2013.12.011

[12] Lautridou C, Lebel B, Burdin G, Vielpeau C (2008) Survival of the cementless Bousquet dual mobility cup: minimum 15-year follow-up of 437 total hip arthroplasties. Rev Chir Orthop Reparatrice Appar Mot 94, 731–739. 19070715 10.1016/j.rco.2008.06.001

[13] Leclercq S, Benoit JY, de Rosa JP, Euvrard P, Leteurtre C, Girardin P (2008) Results of the Evora dual-mobility socket after a minimum follow-up of five years. Rev Chir Orthop Reparatrice Appar Mot 94, 17–22. 10.1016/j.rco.2007.10.015 19070709

[14] Philippot R, Farizon F, Camilleri JP, Boyer B, Derhi G, Bonnan J, Fessy MH, Lecuire F (2008) Survival of cementless dual mobility socket with a mean 17 years follow-up. Rev Chir Orthop Reparatrice Appar Mot 94, e23–e27. 19070710 10.1016/j.rco.2007.10.013

[15] Langlais FL, Ropars M, Gaucher F, Musset T, Chaix O (2008) Dual mobility cemented cups have low dislocation rates in THA revisions. Clinical Orthop Relat Res 466, 389–395. 18196422 10.1007/s11999-007-0047-9 PMC2505133

[16] Merle D’Aubigné R, Postel M (1954) The classic: functional results of hip arthroplasty with acrylic prosthesis. J Bone Joint Surg Am 36-A, 451–475. 13163078

[17] Harris WH (1969) Traumatic arthritis of the hip after dislocation and acetabular fractures: treatment by mold arthroplasty. An end-result study using a new method of result evaluation. J Bone Joint Surg Am 51, 737–755. 5783851

[18] Massin P, Schmidt L, Engh CA (1989) Evaluation of cementless acetabular component migration. An experimental study. J Arthroplasty 4, 245–251. 2795031 10.1016/s0883-5403(89)80020-8

[19] Delee JG, Charnley J (1976) Radiological demarcation of cemented sockets in total hip replacement. Clin Orthop Relat Res 121, 20–32. 991504

[20] Gruen TA, McNeice GM, Amstutz HC. 1979 “Modes of failure” of cemented stem-type femoral components: a radiographic analysis of loosening. Clin Orthop Rela Res 141, 17–27. 477100

[21] Vielpeau C, Lebel B, Ardouin L, Burdin G, Lautridou C (2011) The dual mobility socket concept: experience with 668 cases. Int Orthop 35, 225–230. 21184223 10.1007/s00264-010-1156-8 PMC3032118

[22] Garbuz DS, Masri BA, Duncan CP, Greidanus NV, Bohm ER, Petrak MJ, Della Valle CJ, Gross AE (2012) The Frank Stinchfield Award: Dislocation in revision THA: do large heads (36 and 40 mm) result in reduced dislocation rates in a randomized clinical trial? Clin Orthop Relat Res 470, 351–356. 22038174 10.1007/s11999-011-2146-x PMC3254758

[23] Sanchez-Sotelo J, Haidukewych GJ, Boberg CJ (2006) Hospital cost of dislocation after primary total hip arthroplasty. J Bone Joint Surg Am 88, 290–294. 16452739 10.2106/JBJS.D.02799

[24] Schmalzried TP, Dorey FJ, McKellop H (1998) The multifactorial nature of polyethylene wear in vivo. J Bone Joint Surg Am 80, 1234–1242. 9730133 10.2106/00004623-199808000-00018

[25] Philippot R, Boyer B, Farizon F (2013) Intraprosthetic dislocation: a specific complication of the dual mobility system. Clin Orthop Relat Res 471, 965–970. 23054529 10.1007/s11999-012-2639-2 PMC3563829

[26] Boyer B, Philippot R, Geringer J, Farizon F (2012) Primary total hip arthroplasty with dual mobility socket to prevent dislocation: a 22-year follow-up of 240 hips. Int Orthop 36, 511–518. 21698430 10.1007/s00264-011-1289-4 PMC3291786

[27] Berton C, Girard J, Krantz N, Migaud H (2010) The Durom large diameter head acetabular component: early results with a large-diameter metal-on-metal bearing. J Bone Joint Surg Br 92, 202–208. 20130309 10.1302/0301-620X.92B2.22653

[28] Bin Nasser A, Beaule PE, O’Neill M, Kim PR, Fazekas A (2010) Incidence of groin pain after metal-on-metal hip resurfacing. Clin Orthop Relat Res 468, 392–399. 19862587 10.1007/s11999-009-1133-y PMC2807018

[29] Lefevre C, Wessely L, Dubrana F, Gerard R (2007) The cemented retentive cup: preliminary results at three years. Interact Surgery 2, 165–168.

[30] Shrader MW, Parvizi J, Lewallen DG (2003) The use of a constrained acetabular component to treat instability after total hip arthoplasty. J Bone Joint Surg Am 85-A(11), 2179–2183. 14630850 10.2106/00004623-200311000-00019

[31] Anderson MJ, Murray WR, Skinner HB (1994) Constrained acetabular components. J Arthroplasty 9(1), 17–23. 8163971 10.1016/0883-5403(94)90133-3

[32] Goetz DD, Capello WN, Callaghan JJ (1998) Salvage of total hip instability with constrained acetabular component. Clin Orthop 355, 171–181. 10.1097/00003086-199810000-00018 9917602

[33] Hernigou P, Filippini P, Flouzat-Lachaniette CH, Batista SU, Poignard A (2010) Constrained liner in neurologic or cognitively impaired patients undergoing primary THA. Clin Orthop Relat Res 468, 3255–3262. 20376709 10.1007/s11999-010-1340-6 PMC2974891

[34] Noble P, Durrani S, Usrey M, Mathis K, Bardakos N (2012) Constrained cups appear incapable of meeting demands of revision THA. Clin Orthop Relat Res 470, 1907–1916. 22179979 10.1007/s11999-011-2212-4 PMC3369098

